# Correction to: The Role of Intestinal Stem Cells in Epithelial Regeneration Following Radiation-Induced Gut Injury

**DOI:** 10.1007/s40778-018-0121-0

**Published:** 2018-02-19

**Authors:** Chang-Kyung Kim, Vincent W. Yang, Agnieszka B. Bialkowska

**Affiliations:** 1 Department of Medicine, Stony Brook University School of Medicine, HSC T-17, Rm. 090, Stony Brook, NY 11794 USA; 2 Department of Physiology and Biophysics, Stony Brook University School of Medicine, Stony Brook, NY 11794 USA

**Correction to:** **Curr Stem Cell Rep** **(2017)** **3:320–332**

10.1007/s40778-017-0103-7

The article The Role of Intestinal Stem Cells in Epithelial Regeneration Following Radiation-Induced Gut Injury, written by Chang-Kyung Kim, Vincent W. Yang, and Agnieszka B. Bialkowska, was originally published Online First without open access. After publication in volume 3, issue 4, page 320–332 the author decided to opt for Open Choice and to make the article an open access publication. Therefore, the copyright of the article has been changed to © The Author(s) 2018 and the article is forthwith distributed under the terms of the Creative Commons Attribution 4.0 International License (http://creativecommons.org/licenses/by/4.0/), which permits use, duplication, adaptation, distribution and reproduction in any medium or format, as long as you give appropriate credit to the original author(s) and the source, provide a link to the Creative Commons license, and indicate if changes were made.

